# EV-associated miRNAs from pleural lavage as potential diagnostic biomarkers in lung cancer

**DOI:** 10.1038/s41598-019-51578-y

**Published:** 2019-10-21

**Authors:** Berta Roman-Canal, Cristian Pablo Moiola, Sònia Gatius, Sarah Bonnin, Maria Ruiz-Miró, Esperanza González, Amaia Ojanguren, José Luis Recuero, Antonio Gil-Moreno, Juan M. Falcón-Pérez, Julia Ponomarenko, José M. Porcel, Xavier Matias-Guiu, Eva Colas

**Affiliations:** 1 Department of Pathology and Molecular Genetics/Oncologic Pathology Group, Biomedical Research Institute of Lleida (IRBLleida), University of Lleida, CIBERONC, Lleida, Spain; 2 Department of Pathology, University Hospital of Bellvitge, Bellvitge Biomedical Research Institute (IDIBELL), L’Hospitalet de Llobregat, Barcelona, Spain; 3 Biomedical Research Group in Gynecology, Vall Hebron Research Institute (VHIR), Universitat Autònoma de Barcelona, CIBERONC, Barcelona, Spain; 4 Centre for Genomic Regulation (CRG), The Barcelona Institute or Science and Technology, Dr. Aiguader 88, Barcelona, 08003 Spain; 5 Exosomes Laboratory and Metabolomics Platform. CIC bioGUNE, CIBEREHD Bizkaia Technology Park, Derio, Spain; 6 Department of Thoracic Surgery, Arnau de Vilanova University Hospital, IRBLleida, Lleida, Spain; 7 Gynecological Oncology Department, Vall Hebron University Hospital, CIBERONC, Barcelona, Spain; 8 IKERBASQUE, Basque Foundation for Science, 48011 Bilbao, Spain; 9 Pleural Medicine Unit, Arnau de Vilanova University Hospital, IRBLleida, Lleida, Spain

**Keywords:** Lung cancer, Diagnostic markers

## Abstract

Lung cancer is the leading cause of cancer-related deaths among men and women in the world, accounting for the 25% of cancer mortality. Early diagnosis is an unmet clinical issue. In this work, we focused to develop a novel approach to identify highly sensitive and specific biomarkers by investigating the use of extracellular vesicles (EVs) isolated from the pleural lavage, a proximal fluid in lung cancer patients, as a source of potential biomarkers. We isolated EVs by ultracentrifuge method from 25 control pleural fluids and 21 pleural lavages from lung cancer patients. Analysis of the expression of EV-associated miRNAs was performed using Taqman OpenArray technology through which we could detect 288 out of the 754 miRNAs that were contained in the OpenArray. The differential expression analysis yielded a list of 14 miRNAs that were significantly dysregulated (adj. p-value < 0.05 and logFC lower or higher than 3). Using Machine Learning approach we discovered the lung cancer diagnostic biomarkers; miRNA-1-3p, miRNA-144-5p and miRNA-150-5p were found to be the best by accuracy. Accordance with our finding, these miRNAs have been related to cancer processes in previous studies. This results opens the avenue to the use of EV-associated miRNA of pleural fluids and lavages as an untapped source of biomarkers, and specifically, identifies miRNA-1-3p, miRNA-144-5p and miRNA 150-5p as promising biomarkers of lung cancer diagnosis.

## Introduction

Lung cancer (LC) is the leading cause of cancer-related deaths among men and women in the world, accounting for the 25% of cancer mortality^1^. There are two major forms of LC: non-small cell lung cancer (NSCLC) which is the most common type of LC and include 80% of the cases, and small cell lung cancer (SCLC)^2^. The overall 5-year survival rate of LC is less than 20% mainly due to late diagnosis, whereas patients with tumors diagnosed at early stages have 5-year survival rates of approximately 60%. Diagnosis at early stages of the disease is limited by the fact that LC symptoms occur late in the disease, and current diagnosis rely on the identification of malignant cells from a tissue biopsy^3^. Development of a minimally-invasive diagnosis, based on the identification of sensitive biomarkers in liquid biopsies, could therefore have tremendous impact in decreasing mortality rates with timely therapeutic interventions and disease management.

Several studies have shown the suitability of pleural lavage cytology in early stage surgically resected NSCLC. In these patients, pleural lavage is performed at the beginning of the surgical procedure. Detection of tumor cells in pleural lavage of patients with early stage NSCLC has shown to be associated with shorter overall survival^4^. DNA obtained from pleural lavage material has proven to be appropriate to detect EGFR mutations, even in cases in which tumor cells were not microscopically detected in the lavage^5^. To the best of our knowledge, detection of microRNAs (miRNAs) has not been attempted in this type of material.

MiRNAs are a highly conserved family of small, non-coding RNAs, 19–24 nucleotides in length. They negatively regulate the expression of multiple genes either by including translational silencing or by causing the degradation of messenger RNAs (mRNAs) of the targeted gene, via incomplete base-pairing to a complementary sequence in the 3′-untranslated region (UTR)^6^. MiRNAs are involved in various biologic processes, including cell proliferation, differentiation, death, stress resistances, and fat metabolism; and the aberrant expression of miRNAs has been reported in different diseases and pathological processes including human cancer^2^. miRNAs are detected in tumor tissues but also in body fluids, including extracellular vesicles (EVs). EVs are 20–200 nm round membrane vesicles released by multivesicular bodies fusing with the cell membrane. Their principal function is to participate in the intercellular communication and because of their content in bioactive material such proteins, metabolites, RNA and miRNAs, EVs have been considered an important source of biomarkers for the scientific community. This material is well-protected owing to the EVs lipid bilayer membrane, even if EVs are extracted from circulating or proximal body fluids^7^.

To date, several studies have shown the promising role of exosomal miRNAs as diagnostic biomarkers of LC in plasma^8,9^. Rabinowits *et al*. in 2009 identified a profile of 12 miRNAs which were increased in both tissue and circulating exosomes of NSCLC patients compared to controls, demonstrating that exosomal miRNA can accurately reflect the tumor profile in the absence of tumor tissue^10^. More recently, Giallombardo *et al*.^11^ unveiled 8 miRNAs that were deregulated in NSCLC comparing to healthy donors and, Jin *et al*. developed a miRNA profile of 4 miRNAs that exhibited sensitivity of 80.25% and specificity of 92.31% with an AUC value of 0.899 for diagnosing 43 NSCLC patients over 60 controls^12^. Nevertheless, any of those biomarkers have reached the clinical practice, probably due to lack of validation.

New approaches focusing on proximal fluids, i.e. fluids in direct or close contact with the tumor, might provide higher sensitivity and specificity to diagnose LC. Herein, we investigated the use of EVs isolated from the pleural lavage, a proximal fluid in LC patients, as a source of potential diagnostic biomarkers. We conducted miRNA-profiling of EVs isolated from pleural lavages from surgical LC patients, specifically from adenocarcinoma lung cancer (ADC) and lung squamous carcinoma (LUSC) patients, and we unveiled the most relevant individual miRNAs for diagnosing LC. We used a series of non-cancer patients with pleural effusion as a control. The study was conceived as a proof of concept investigation to demonstrate the feasibility of pleural lavage as a source of EV-associated miRNAs in patients with LC.

## Results

**Figure 1:**
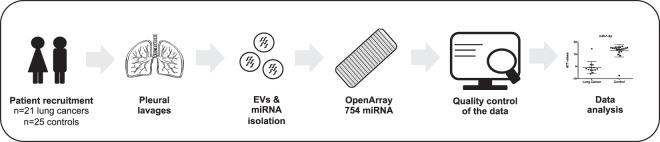
Workflow. Workflow of the study design.

We analyzed the miRNA profile of EVs isolated from the pleural fluids and lavages of 46 patients, including 25 control and 21 LC patients. Figure 1 illustrates the workflow that was followed in this study. Quality of EVs isolated from the pleural fluids and lavages was measured by size distribution and concentration by nanoparticle Tracking analysis, immunoblot and electron microscopy (Supplementary Fig. S1). miRNAs were extracted from EVs for a systematic miRNA expression analysis using the Taqman OpenArray technology through which we could detect 288 out of the 754 miRNAs that were contained in the OpenArray. The quality of the data included the removal of probes that had a Ct value of 40 in all samples, and the removal of samples in which more than 80% of the probes had a Ct value above 40. Finally, a total of 272 miRNA were kept for the differential expression analysis of 20 control and 14 LC patients (Table 1).

**Table 1:** Clinicopathological characteristics of patients.

	Lung Cancer	Control
**Age**		
Median	68	77
Minimum	52	62
Maximum	84	92
**Gender**		
Female	2	9
Male	12	11
**Pathology**		
Lung Cancer	14	—
ADC	9	—
LUSC	7	—
Heart failure	—	14
Hepatic hydrothorax	—	2
Post CABG surgery	—	1
Constructive pericarditis	—	1
SVC obstruction	—	1
Chronic kidney disease	—	1

Clinical characteristics of the final cohort of patients included in the study after data normalization.

*ADC: adenocarcinoma.

*LUSC: lung squamous carcinoma.

*SVC: superior vena cava.

*CABG: coronary artery bypass graft.

**Figure 2:**
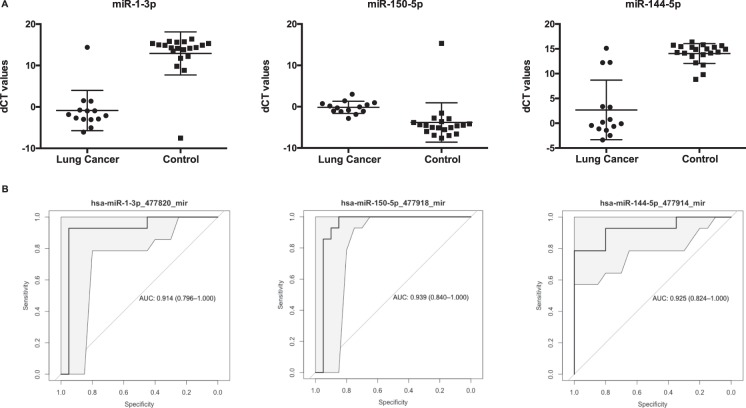
Diagnostic performance of the top differentially expressed miRNAs. (**A**) Relative dCT values of top differentially expressed miRNAs (miRNA-1-3p, miRNA-150-5p, and miRNA-144-5p) in patients with lung cancer (n = 14) compared to control patients (n = 20). **p < 0.05. (**B**) ROC-curves and AUC-scores for miRNA-1-3p, miRNA-150-5p, and miRNA-144-5p.

The differential expression analysis between cancer and control cases yielded a list of 14 miRNAs that were significantly dysregulated (adj. p-value < 0.05 and logFC lower or higher than 3). Among those, 5 miRNA were found to be upregulated and 9 were downregulated in LC patients (Table 2; Supplementary Fig. S2). In order to evaluate whether differential expression translated into diagnostic power, we perform a predictive analysis with all the differentially expressed miRNAs. The logistic model was repeated 500 times to assess the model reproducibility in a divided cohort of training and validation set following a 2:1 ratio; and then the classification performance was evaluated in the whole cohort (Table 3). The best classifier was miRNA-1-3p, which showed an average accuracy of 0.941 (95% CI: 0.803–0.993), sensitivity of 0.929, specificity of 0.950 and AUC value of 0.914. MiRNA-1-30p presented a 13-fold expression, which was lower in LC patients than in controls (adj. p-value of 1.92e-04). The next best classifiers, miRNA-144-5p and miRNA-150-5p, showed an average AUC values comparable with that of miRNA-1-30p with, however, significantly lower accuracy (0.882 and 0.912, respectively) and sensitivity (0.786 and 0.857) for the same specificity. miRNA-144-5p presented a 11-fold expression which was also lower in LC patients than in controls (adj. p-value of 1.28e-02) while miRNA-150-5p presented an expression higher in LC patients with a 3-fold expression (adj. p-value of 3.91e-02) (Fig. 2).

**Table 2:** miRNA transcripts displaying a significant differential expression in patients with lung cancer compared to control patients.

ID	logFC	P-Value	adj.P-Va
hsa-miR-150-5p_477918_mir	3,65	1,87E-03	3,91E-02
hsa-miR-144-5p_477914_mir	−11,37	3,76E-04	1,28E-02
hsa-miR-1-3p_477820_mir	−13,78	1,41E-06	1,92E-04
hsa-miR-584-5p_478167_mir	−9,55	1,90E-08	5,17E-06
hsa-miR-133b_480871_mir	−7,72	1,30E-03	3,52E-02
hsa-miR-451a_478107_mir	−3,29	3,50E-04	1,28E-02
hsa-miR-27a-5p_477998_mir	4,93	8,05E-05	7,30E-03
hsa-miR-21-3p_477973_mir	5,65	1,99E-04	1,28E-02
hsa-miR-199a-5p_478231_mir	−7,73	3,47E-04	1,28E-02
hsa-miR-1249-3p_478654_mir	6,70	5,62E-04	1,70E-02
hsa-miR-485-3p_478125_mir	4,80	1,82E-03	3,91E-02
hsa-miR-20b-5p_477804_mir	−8,18	3,05E-04	1,28E-02
hsa-miR-181c-5p_477934_mir	−3,79	2,06E-03	4,01E-02
hsa-miR-30e-5p_479235_mir	−6,46	1,45E-03	3,59E-02

Log fold-change expression, p-value and adjusted p-value of the 14 miRNAs significantly dysregulated in EVs from the pleural lavage of lung cancer patients compared to control (adj. p-value < 0.05 and logFC lower or higher than 3).

**Table 3:** Performance of the top diagnostic miRNA biomarkers.

miRNA	AUC	Accuracy	95% CI	Sensitivity	Specificity
**Logistic model on the 2:1 cohort**					
hsa-miR-1-3p_477820_mir	0,923	0,941	[0.936; 0.946]	0,938	0,943
hsa-miR-144-5p_477914_mir	0,925	0,878	[0.872; 0.883]	0,776	0,941
hsa-miR-150-5p_477918_mir	0,937	0,825	[0.818; 0.831]	0,666	0,925
**Logistic model on the whole cohort**					
hsa-miR-1-3p_477820_mir	0,914	0,941	[0.803; 0.993]	0,929	0,95
hsa-miR-150-5p_477918_mir	0,939	0,912	[0.763; 0.981]	0,857	0,95
hsa-miR-144-5p_477914_mir	0,925	0,882	[0.725; 0.967]	0,786	0,95

AUC values, accuracy, sensitivity, specificity and 95% of confidence intervals are summarized for those miRNAs which were selected for the highest diagnostic performance on the 2:1 cohort (on top) and the whole cohort (below).

**Figure 3:**
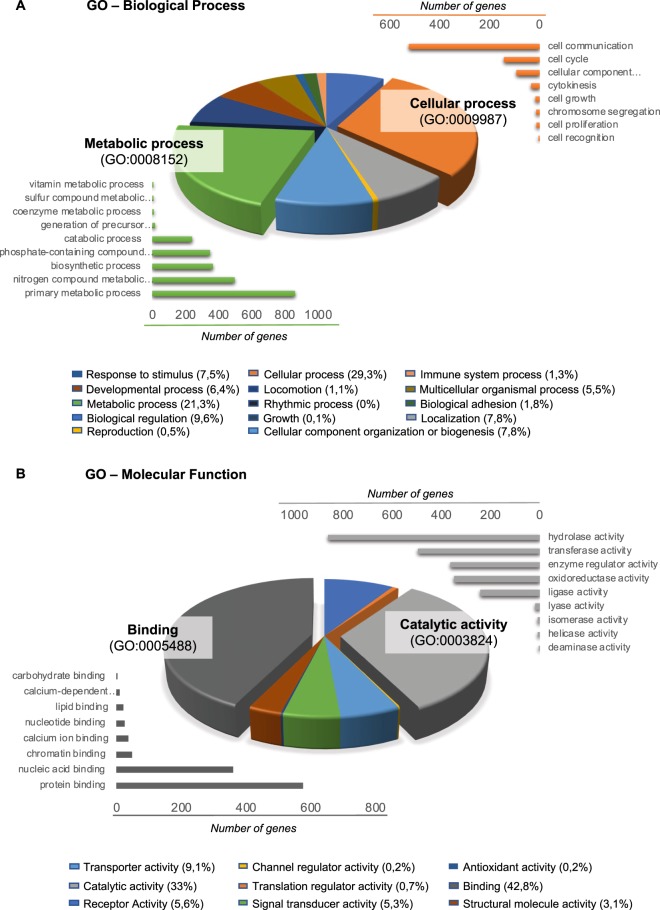
GO terms associated to the predicted proteins regulated by the differentially expressed miRNAs in lung cancer and control patients. (**A**) GO analysis of up-regulated and down-regulated target genes according to biological process. (**B**) GO analysis of up-regulated and down-regulated target genes according to molecular function.

In order to further understand the tumor biology related to the specific EV-associated miRNA content of LC patients, we performed a bioinformatics study to first, unveil the proteins that are regulated by the differential miRNAs, and then, assess their biological and molecular function. A total of 3,745 proteins were found to be regulated by the differential miRNA, specifically 812 proteins were associated to the 5 overexpressed miRNA whilst 2,933 proteins were controlled by the 9 downregulated miRNA (Table 4). To comprehensively integrate the properties of all target proteins, these were studied using Gene Ontology (GO). The most enriched biological functions in LC EVs were cellular processes (29.3%), mostly including cell communication, cell cycle and cellular component movement; and metabolic processes (21.3%), including primary metabolic process, nitrogen compound metabolic process and biosynthetic process (Fig. 3A). In relation to the most altered molecular functions in LC EVs, the Gene Ontology (GO) analysis revealed that many targeted proteins were found to be involved in binding (42.8%), including protein and nucleic acid binding; and in catalytic activity (33%), including hydrolase and transferase activity (Fig. 3B).

**Table 4:** Prediction of miRNA target proteins.

miRBASE code	ID	logFC_A_vs_B	# Proteins regulated*
**5 miRNA up-regulated - target 812 unique proteins**			
MIMAT0000451	hsa-miR-150-5p_477918_mir	3,65	518
MIMAT0002176	hsa-miR-485-3p_478125_mir	4,80	247
MIMAT0004501	hsa-miR-27a-5p_477998_mir	4,93	33
MIMAT0004494	hsa-miR-21-3p_477973_mir	5,65	55
MIMAT0005901	hsa-miR-1249-3p_478654_mir	6,70	9
**9 miRNA down-regulated - 2933 unique proteins**			
MIMAT0000416	hsa-miR-1-3p_477820_mir	−13,78	460
MIMAT0004600	hsa-miR-144-5p_477914_mir	−11,37	19
MIMAT0003249	hsa-miR-584-5p_478167_mir	−9,55	269
MIMAT0001413	hsa-miR-20b-5p_477804_mir	−8,18	1060
MIMAT0000231	hsa-miR-199a-5p_478231_mir	−7,73	364
MIMAT0000770	hsa-miR-133b_480871_mir	−7,72	429
MIMAT0000692	hsa-miR-30e-5p_479235_mir	−6,46	662
MIMAT0000258	hsa-miR-181c-5p_477934_mir	−3,79	865
MIMAT0001631	hsa-miR-451a_478107_mir	−3,29	13

The proteins regulated by each miRNA was predicted using the Predictive Target Module of miRWalk 2.0 sofware and minimized to those that were found in at least 8 out of 12 databases. The total number of predicted proteins is plotted for each dysregulated miRNA.

## Discussion

In this study, we analyze the EV-associated miRNA profiles of 25 control pleural fluids and 21 pleural lavages from LC patients by using the Taqman OpenArray technology. The differential expression analysis between the two groups yielded a list of 14 miRNAs that were significantly dysregulated, and among them, the best diagnostic biomarkers were miRNA-1-3p, miRNA-144-5p and miRNA-150-5p with an accuracy to label diagnose LC of 0.941, 0.882 and 0.912, respectively.

In our study, miR-1-3p was found to be downregulated by a 13-fold expression in LC patients compared to controls (adj. p-value of 1.92e-04). This is in accordance to observations by other groups, in which miR-1-3p was identified as a tumor-suppressed miRNA in different types of cancer such as prostate^13,14^, liver^15^ and bladder^16^. MiR-1-3p suppressed proliferation, invasion and migration of bladder cancer cells by up-regulating SFRP1 expression^17^. In LC, Nasser *et al*. showed that miR-1 expression is reduced in LC and inhibits the tumorigenic potential of LC cells by down-regulating oncogenic targets, such as MET and FoxP1^18^.

Also in accordance with our finding that miR-144-3p is 11-fold times downregulated in LC, many studies in several types of cancers have reported that miR-144-3p acted as an antitumor miRNA^19,20^ and, recently, it has been reported that both strands of miR-144-5p and miR-144-3p showed a significantly downregulated expression in renal cell carcinoma (RCC) tissues and that they functioned as tumor suppressors in RCC cells^21^ and bladder cancer^22^. In LC, miR-144-5p was found to be downregulated in NSCLC clinical specimens as well as in NSCLC cell lines exposed to radiation suggesting that deregulation of the miR-144-5p plays an important role in NSCLC cell radiosensitivity, thus representing a new potential therapeutic target for NSCLC^23^. A recent study revealed that miR-144-5p and miR-451a inhibited cell proliferation^24^.

MiRNA-150-5p was upregulated by a 3-fold expression in LC patients. In other studies of LC, miRNA-150-5p was found to be upregulated in tissue suggesting that miRNA-150-5p may be involved in the pathogenesis of LC as an oncogene^25–27^. However, studies in other types of cancer, i.e. glioma^28^, cholangiocarcinoma (CCA)^29^ and colon cancer^30^, showed a tumor suppressor role of miRNA-150-5p. *In vitro* experiments on regulation of CCA found that miR-150-5p overexpression inhibited tumor cell proliferation, migration, and invasion capacity, whereas knockdown of miR-150-5p expression induced tumor cell proliferation, migration, and invasion^29^. In colorectal cancer tissues, decreased miR-150-5p was found to be associated with poor overall survival^31^.

In the clinical setting, our study provides the evidence that the use of EV-associated miRNA isolated from pleural fluids and lavages are a potential source of biomarkers for LC. Most of the studies use plasma as it is the most common, easy-to-handle, accessible liquid biopsy. However, the use of proximal fluids offers an improved representation of the molecular alterations that takes place in the tumor. Hence, although proximal fluids, such as the pleural fluid, may occasionally be more difficult to obtain, they might serve as a powerful tool to identify biomarkers for lung-related diseases. In relation to proximal fluids related to LC, studies performed by Admyre *et al*.^32^ and more recently, Ji Eun Kim *et al*.^33^ highlighted the use of another type of fluid, i.e. the bronchoalveolar lavage (BAL). Although this fluid is obtained in a non-invasive manner, biomarkers identified in BAL might only represent tumors localized within the lung and/or in direct contact with the airway. Nevertheless, pleural lavages are expected to provide biomarkers from tumors localized in different sites, i.e. inside and outside of the lungs. Importantly, our study unveiled the promising use of miRNA-1-3p, miRNA-144-5p and miRNA-150-5p as diagnostic biomarkers. Those biomarkers should be validated as well as combined in order to increase the already excellent accuracy of the individual miRNA. However, this should be done in an independent study including a larger cohort of patients and controls. Interestingly, mesothelioma patients might also be compared to LC patients in future studies. Moreover, further analysis should be performed to elucidate the prognostic value of the detection of the different types of miRNAs in EVs isolated from pleural lavages.

## Conclusion

In this work, we have demonstrated that use of EV-associated miRNA of pleural fluids and lavages are an untapped source of biomarkers, and specifically, we identified miRNA-1-3p, miRNA-144-5p and miRNA 150-5p as promising biomarkers for LC diagnosis.

## Methods

### Patients and pleural fluid and lavages collection

A total of 46 participants were recruited at Hospital Arnau de Vilanova in Lleida, Spain. All the patients participating signed an informed consent and the study was approved by the Clinical Research Ethics Committee of the hospital. All experiments were performed in accordance with relevant guidelines and regulations of the hospital. Pleural fluids and lavages were extracted from a cohort of 46 patients, corresponding to 25 control patients with benign pleural effusions, and 21 patients with ADC or LUSC, who underwent curative surgery. In control patients, the collection of pleural fluid was performed under local anesthesia (2% mepivacaine) by the introduction of a metallic needle in the pleural cavity through an intercostal space. The pleural fluid was gently aspirated, collected in a 50 mL tube and stored at −80 °C. In LC patients, the pleural lavage was collected, during surgery, after accessing the thoracic cavity and prior to any manipulation of the lung. A total of 100 cc of physiological saline were instilled into the pleural cavity with a 50 cc syringe, mobilizing the patients for its correct distribution of the serum and were extracted with a 50 cc syringe connected to a 14-gauge aspiration needle. A volume ranging from 80 to 90 mL was collected in 50 mL tubes and stored at −80 °C. All fluids were non-hemorrhagic and proved to be exudates. The clinical features of each patient are listed in Supplementary Table S1. The diagnosis of LC was based on cytohistological background, while that of benign pleural effusions relied on well-established clinical criteria.

### EVs isolation

EVs were isolated with a differential centrifugation method, following a modification of a previously described EVs isolation protocol^34^. Pleural fluids and lavages were centrifuged by Thermo Scientific Heraeus MultifugeX3R Centrifuge (FiberLite rotor F15-8 × −50c) at 300 × g during 10 min, followed by a centrifugation step at 2500 × g during 20 min and a centrifugation step at 10,000 g during 30 min. After, the supernatant was filtered through 0.22 µm filters (Merck Millipore) and the sample obtained was transferred to ultracentrifuge tubes (Beckman Coulter) and filled with PBS. To finish the centrifuged procedure, two consecutive ultracentrifugation steps at 100,000 g were performen on a Thermo Scientific Sorvall WX UltraSeries Centrifuge with an AH-629 rotor during 2 hours each. At the end, the pellet obtained with the EVs was resuspended in 50 µL of PBS. From those, 5 µL were isolated for nanoparticle tracking analysis (NTA) and quantification, and the rest was frozen at −80 °C with 500 µL of Qiazol for RNA extraction, or with 45 µL of RIPA buffer (5 nM EDTA, 150 mM NaCl, 1% Triton, 20 nM Tris pH8 and 1:200 protein inhibitors) for protein extraction.

### Nanoparticle tracking analysis

NTA was performed using a Nanosight LM10 instrument equipped with a 405 nm laser and a Hamamatsu C11440 ORCA-Flash 2.8 camera (Hamamatsu) with Nanoparticle Tracking Analysis (NTA, Malvern Instruments, UK) and data was analyzed with the NTA software 2.3 following the manufacter’s instructions. To define the size and concentration of the particles, the samples were diluted appropriately with Milli-Q water (Milli-Q Synthesis, Merck Millipore, Massachusetts, USA) to give counts in the linear range of the instrument. The particles in the laser beam undergo Brownian motion, and a video was recorded for 60 s in triplicate.

### Immunoblot

Protein extracts of EVs were obtained by unfrozen the RIPA-containing EVs samples, incubating for 1 h at 4 °C, and sonication. Protein extracts were loaded and separated by a 10% SDS-PAGE and transferred to PVDF membranes. For blocking, membranes were soaked in 5% non-fat dried milk in TBS-Tween20 (0.01%). Proteins were immunodetected using primary antibodies: mouse anti-CD9 (1:250; ref. 555370, BD Biosciences) and mouse anti-TSG101 (1:500; ref. ab83, Abcam). For the incubation with a secondary HRP-coupled antibody (rabbit anti-mouse Immunoglobulins/HRP, 1:2000, ref. P0260, Dako), PVDF membranes were firstly washed and then the incubated was performed. Finally, we revealed using the Immobilon Western Chemiluminiscent HRP Substrate (ref. WBKLS0100; Merck Millipore) and the intensity of the bands was quantified using the ImageJ software (v. 1.45 s).

### Electron microscopy

For cryo-electron microscopy, EV preparations were directly adsorbed onto glow-discharged holey carbon grids (QUANTIFOIL, Germany). Grids were blotted at 95% humidity and rapidly plunged into liquid ethane with the aid of a VITROBOT (Maastricht Instruments BV, The Netherlands). Vitrified samples were imaged at liquid nitrogen temperature using a JEM-2200FS/CR transmission cryo-electron microscope (JEOL, Japan) equipped with a field emission gun and operated at an acceleration voltage of 200 kV.

### Total RNA extraction

The total RNA was isolated from the EVs samples containing Qiazol by using the miRNeasy MiniKit (Qiagen) and following the manufacturers’ protocol. RNA from EVs was eluted with 30 µL of Nuclease-free water (Ambion) and then were stored at −80 °C for their future utilization.

### Openarray analysis

miRNA expression was performed using a fixed-content panel containing 754 well-characterized human miRNA sequences from the Sanger miRBase v14 (Catalog number: 4470187, Thermo Fisher Scientific) following the procedure of previous studies of the group^35,36^. Reverse transcription (RT) was performed on 2 µL RNA using Megaplex™ Primer Pools A and B and the supporting TaqMan® MicroRNA Reverse Transcription Kit as follows: 15 min at 42 °C and 5 min at 85 °C. Then, 5 uL of the resulting cDNA was preamplified prior to real-time PCR analysis using Megaplex™ PreAmp Pools and the TaqMan® PreAmp Master Mix using the following conditions: one single step at 95 °C during 5 min, 20 cycles of a two-steps program (3 sec, 95 °C and 30 sec, 60 °C) followed by a single cycle of 10 min at 99 °C to inactivate the enzyme. The preamplified products were diluted 1:20 in 0.1x TE buffer pH8.0, and mixed in 1:1 with TaqMan® OpenArray® Real-Time PCR Master Mix in the 384-well OpenArray® Sample Loading Plate. TaqMan® OpenArray® MicroRNA Panels were automatically loaded using the AccuFill™ System.

### Preprocessing and differential expression analysis

The bioinformatics analysis was performed with the BioConductor (version 3.7)^37^ project in the R statistical environment (version 3.5.0) **[**R Core Team (2015): R: A language and environment for statistical computing. R Foundation for Statistical Computing, Vienna, Austria. http://www.R-project.org/**]**. HTqPCR (version 1.34) R package^38^ was used to proceded the data. Probes that had a “Cycle threshold” (Ct) value of 40 in all samples were removed. Further samples in which more than 80% of the probes had a Ct value above 40 were retained. To assure comparability across samples, the Ct values were delta normalized. The average of the probes *hsa−miR−324−5p, hsa−miR−128−3p, hsa−miR−24−3p*, and *hsa−miR−148a−3p* were used for normalization of the Ct values. Those probes were selected based on having Ct value of 40 in a maximum of three samples, and the lowest interquartile range across samples. Differential expression analysis was carried out with an empirical Bayes approach on linear models, using the limma (version 3.36) R Package^39^. Results were corrected for multiple testing using the False Discovery Rate (FDR)^40^.

### Development of predictors

The whole patient cohort was divided into training and validation sets with the 2:1 ratio for predictive analysis. Calculated (with limma) relative miRNA expression values were used as input variables to a logistic regression model between groups. Each significant (adj. p-value < 0.05) deregulated miRNA was fitted into the logistic regression model to differentiate the LC and the control patient’s groups; and the model classification performance was evaluated using the AUC (area under the ROC curve), accuracy, sensitivity and specificity values on the validation set. The procedure of partitioning the dataset into training and validation sets and fitting the logistic model was repeated 500 times to assess the model reproducibility and collect statistics. Finally, AUC values for each selected predictor were calculated in the whole cohort.

### Prediction of miRNA target genes and bioinformatics analysis

Predicted miRNAs target genes were obtained using the Predictive Target Module of miRWalk2.0 online software^41^ (https://goo.gl/ajG9ja). To improve the accuracy of target gene prediction and reduce the rate of false positives, we considered as valid target genes only those transcripts that were predicted in at least 8 out of the 12 databases (miRWalk, miRanda, MicroT4, miRDB, miRMap, miRBridge, miRNAMap, PICTAR2, RNA22, PITA, TargetScan, and RNAhybrid). To analyze the potential functions of the predicted target genes, we performed a Gene Ontology (GO) functional analysis using the online Panther software^42^ (http://www.pantherdb.org/). Biological process (BP) and molecular function (MF) GO terms were analyzed and plotted.

## Supplementary information

Supplementary Information
