# Supplementary material for: EV-associated miRNAs from pleural lavage as potential diagnostic biomarkers in lung cancer
Source: Sci Rep. 2019 Oct 21;9:15057. doi: 10.1038/s41598-019-51578-y (PMC6803646; doi:10.1038/s41598-019-51578-y)
Supplement: Supplementary file 1 — Supplementary Information [file 41598_2019_51578_MOESM1_ESM.pdf]

EV-associated miRNAs from pleural lavage as potential diagnostic biomarkers in lung cancer

Berta Roman-Canal[1,2]; Cristian Pablo Moiola[1,3]; Sònia Gatius[1]; Sarah Bonnin[4]; Maria Ruiz-Miró[1]; Esperanza González[5]; Amaia Ojanguren[6]; José Luis Recuero[6]; Antonio Gil-Moreno[3,7]; Juan M. Falcón-Pérez[5,8]; Julia Ponomarenko[4]; José M. Porcel[9]*; Xavier Matias-Guiu[1,2]* and Eva Colas[3]*

Supplementary Figure - 1

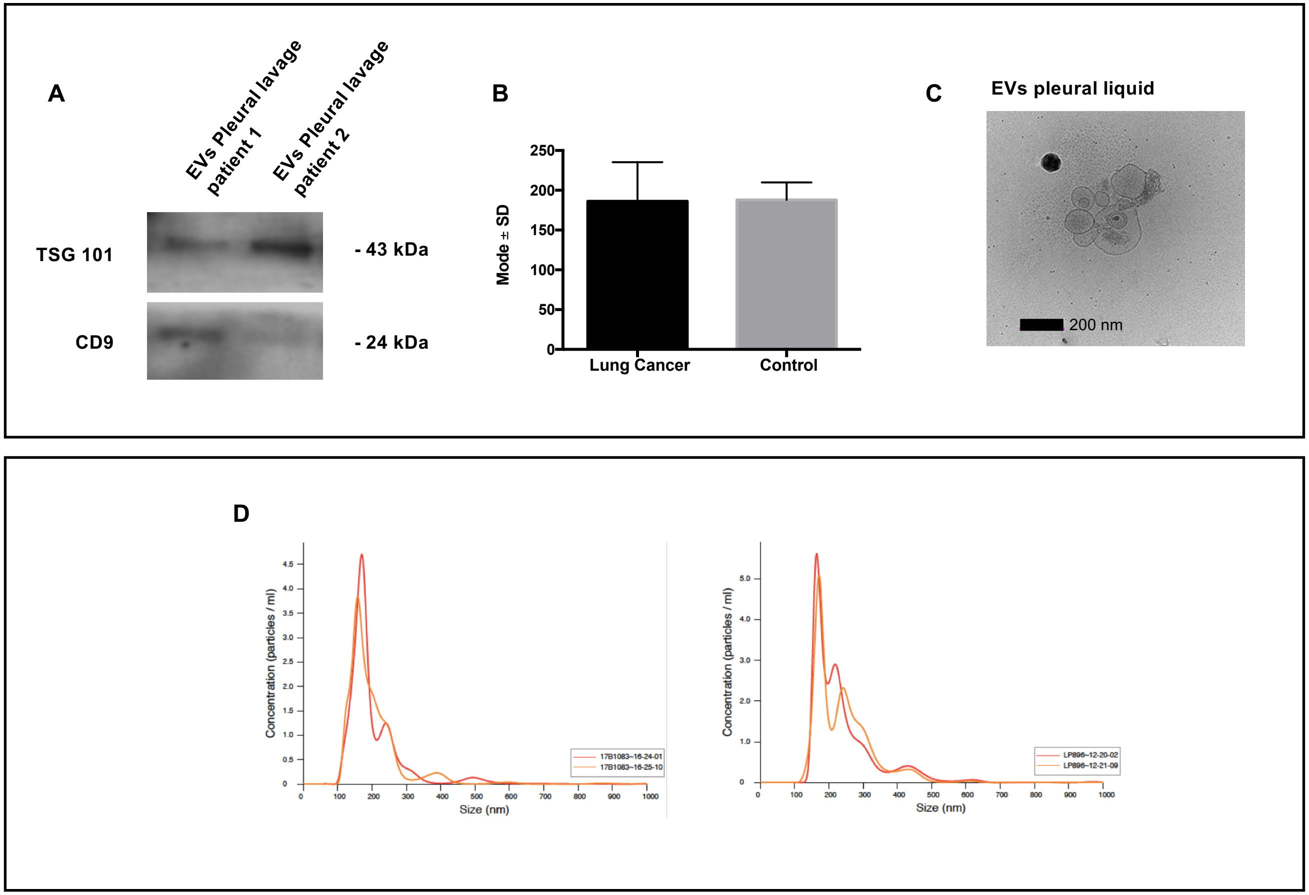

Supplementary Figure - 2

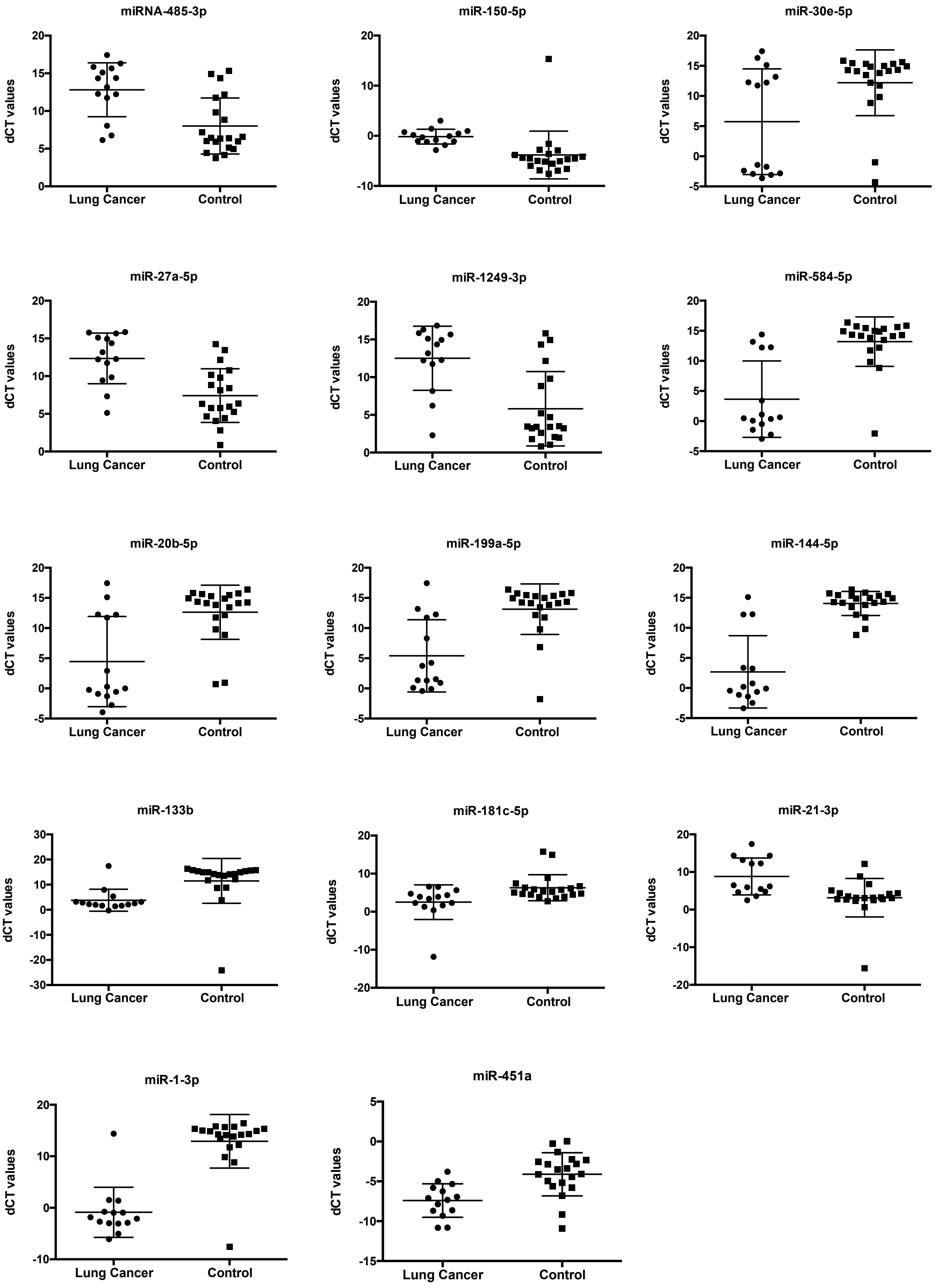

Supplementary Table - 1

Patient	Pathology	Age	Gender	Histological diagnostic	Histological grade	Stage	Metastasis
1	Hepatic hydrothorax	71	Female				
2	Heart failure	88	Male				
3	SVC obstruccion	92	Female				
4	Hepatic hydrothorax	62	Male				
5	Heart failure	78	Female				
6	Heart failure	90	Female				
7	Heart failure	71	Female				
8	Post CABG surgery	77	Male				
9	Heart failure	89	Female				
10	Heart failure	89	Male				
11	Constructive pericarditis	64	Male				
12	Pneumonia	88	Male				
13	Heart failure	89	Female				
14	Heart failure	91	Female				
15	Heart failure	85	Male				
16	Heart failure	92	Male				
17	Heart failure	91	Female				
18	Heart failure	94	Female				
19	Heart failure	90	Female				
20	Heart failure	84	Male				
21	Heart failure	90	Male				
22	Heart failure	78	Male				
23	Heart failure	80	Male				
24	Heart failure	90	Female				
25	Chronic kidney disease	85	Male				
26	Lung Cancer	52	Female	ADC	-	T2a N2 Mx	No
27	Lung Cancer	57	Male	LUSC	3	T2a N0 Mx	No
28	Lung Cancer	54	Male	ADC	3	T2b N0 M1	Brain
29	Lung Cancer	49	Female	ADC	1	T1a N0 Mx	No
30	Lung Cancer	66	Male	LUSC	2	T1a N0 Mx	No
31	Lung Cancer	59	Male	LUSC	2	T1b N0 Mx	No
32	Lung Cancer	64	Male	LUSC	2	T2a N1 Mx	No
33	Lung Cancer	68	Male	ADC	2	T1b N0 Mx	No
34	Lung Cancer	57	Male	ADC	3	T2a N2 Mx	No
35	Lung Cancer	67	Male	LUSC	2	T1a N0 Mx	No
36	Lung Cancer	61	Male	ADC	2	T1a N0 Mx	No
37	Lung Cancer	56	Male	LUSC+ADC	1+2	T1c Nx; T2a Nx	No
38	Lung Cancer	56	Male	ADC	-	T4 N0 M1	Vertebral
39	Lung Cancer	65	Male	ADC	1	T2b N0 Mx	No
40	Lung Cancer	65	Male	LUSC+ADC	1+2	T1a N0 Mx; T1a N0 Mx	No
41	Lung Cancer	84	Male	LUSC	2	T2a N0 Mx	No
42	Lung Cancer	57	Male	ADC	2	T2a N0 Mx	No
43	Lung Cancer	62	Female	LUSC	1	T1a N0 Mx	No
44	Lung Cancer	60	Female	ADC	1	T2a N0 Mx	No
45	Lung Cancer	77	Male	ADC	2	T2a N0 Mx	No
46	Lung Cancer	75	Male	ADC	3	T1b N2 Mx	No

* ADC: adenocarcinoma
* LUSC: lung squamos carcinoma
* G1, G2 and G3: Grade 1, 2 and 3
* N0, N1 and N2: adenopaties afection
* Mx: no metastasis were seen by the pathologist
SVC: superior vena cava
CABG: coronary artery bypass graft

SUPPLEMENTARY LEGENDS

Supplementary Figure S1. EVs characterization. (**A**) Cropped immunoblot against TSG101 and CD9 in representative samples of EVs isolated from the pleural lavage of two lung cancer patients. (**B**) Box-plot representing the average mode of EVs isolated from the pleural lavage and fluid of lung cancer and control patients, respectively (Mean ± SD); measured by Nanoparticle Tracking Analysis. (**C**) Electron microscopy image of EVs isolated from pleural liquid of a control patient. (**D**) Size distribution and concentration of isolated EVs of a pleural lavage of a lung cancer patient (left) and a pleural fluid of a control patient (right), measured by Nanoparticle Tracking Analysis.

Supplementary Figure S2. Box-plots of all the differentially expressed miRNAs. Box-plots of the 14 miRNAs that were significantly dysregulated in EVs from the pleural lavage of lung cancer patients (n=14) compared to the pleural fluid of control patients (n=20).

Supplementary Table S1. Clinicopathological characteristics of all patients. Clinical characteristics of the total cohort of patients recruited in the study.
